# Artificial Pneumothorax Using the Liver-Directed Approach and Seldinger’s Technique: Technical Feasibility and Safety

**DOI:** 10.7759/cureus.41423

**Published:** 2023-07-05

**Authors:** Mizuki Ozawa, Miyuki Sone, Shunsuke Sugawara, Chihiro Itou, Shintaro Kimura, Yasuaki Arai, Masahiko Kusumoto

**Affiliations:** 1 Diagnostic Radiology, National Cancer Center Hospital, Tokyo, JPN

**Keywords:** percutaneous ablation, percutaneous biopsy, complication, interventional radiology, artificial pneumothorax

## Abstract

Purpose

This study aimed to evaluate the technical feasibility and safety of artificial pneumothorax induction for percutaneous procedures using the liver-directed approach and Seldinger’s technique.

Materials and methods

The data of 25 consecutive patients who underwent percutaneous procedures after inducing artificial pneumothorax were reviewed retrospectively. The liver surface was punctured with an 18-gauge indwelling needle via the intercostal space in the inferior thoracic cavity under ultrasound guidance, avoiding the lung parenchyma and leaving the catheter in place. After a deep inhalation pulled the catheter tip into the pleural cavity, a hydrophilic guidewire was inserted through the catheter. Finally, a small-diameter catheter was inserted into the pleural cavity over the guidewire to induce artificial pneumothorax. Procedure time (the time from local anesthesia to completion of the procedure), technical success (successful induction of artificial pneumothorax), clinical success (successful completion of the percutaneous procedure), and complications (categorized according to the Clavien-Dindo classification) were evaluated in this study.

Results

The artificial pneumothorax induction was successful in all cases. Clinical success was achieved in 23 of 25 procedures (92%). No severe complications were observed.

Conclusion

The liver-directed approach and Seldinger’s technique for inducing artificial pneumothorax was safe and feasible for avoiding lung injury.

## Introduction

The key to avoiding complications associated with percutaneous needle biopsy and ablation is to limit injury to other organs. The lung may be involved in the puncture route during the procedures for mediastinal and subdiaphragmatic liver tumors. A multicenter retrospective study of 9,783 percutaneous lung biopsies reported 10 cases (0.10%) of tension pneumothorax, nine cases (0.092%) of hemothorax, and six cases (0.061%) each of air embolism and severe pulmonary hemorrhage or hemoptysis [1]. Lung biopsy and percutaneous procedures traversing the lung parenchyma may have similar severe adverse events. 

Artificial pneumothorax, originally described by Forlanini as a treatment for tuberculosis more than 100 years ago, has recently been proposed to avoid lung injury during percutaneous procedures [2]. The techniques for inducing artificial pneumothorax vary in the literature; however, most studies applied the one- or two-step methods. The first step is to inject a small amount of gas by puncturing the pleural cavity with a fine needle; in the two-step method, the space created by the needle is then punctured with another needle [3-5]. Although both methods are technically simple, the two-step method requires two punctures, and both may cause lung injuries. Furthermore, deflation after the procedure may be challenging because only a fine needle is used.

The method of inducing artificial pneumothorax by introducing a fine needle into the liver surface in the inferior region of the thoracic cavity, avoiding the lung parenchyma, and using a guidewire is safe and commonly performed in clinical practice. Therefore, we named this method of creating artificial pneumothorax the liver-directed approach and Seldinger’s technique. This retrospective study aimed to evaluate the technical feasibility and safety of inducing artificial pneumothorax for percutaneous procedures using this technique.

## Materials and methods

Patients

Institutional review board approval was obtained for this retrospective study, and the requirement for informed consent was waived. However, all patients provided written informed consent to undergo the procedure. The data of 25 consecutive patients (17 males and eight females; mean age 70.8 years) who underwent percutaneous procedures with artificial pneumothorax induction between 2013 and 2021 were reviewed. The procedures included 15 radiofrequency ablations (RFA) for hepatocellular carcinoma, eight biopsies for hepatocellular carcinoma, mediastinal tumor and adrenal tumor, and two cryotherapies for renal cell carcinoma.

Technique

**Figure 1:**
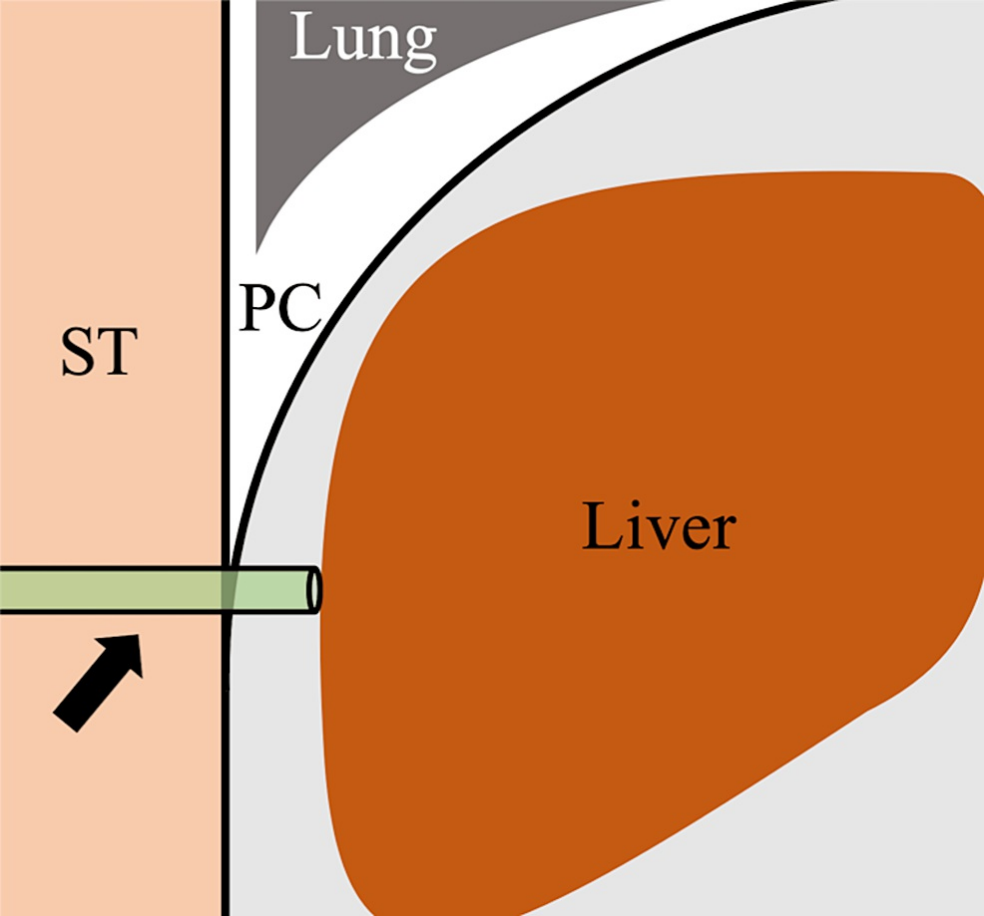
Schema of Seldinger’s technique to induce artificial pneumothorax (coronal image) The liver surface is punctured using an 18-gauge indwelling needle via the pleural cavity under ultrasound guidance. The catheter is left in place (black arrow). ST: subcutaneous tissue, PC: pleural cavity.

**Figure 2:**
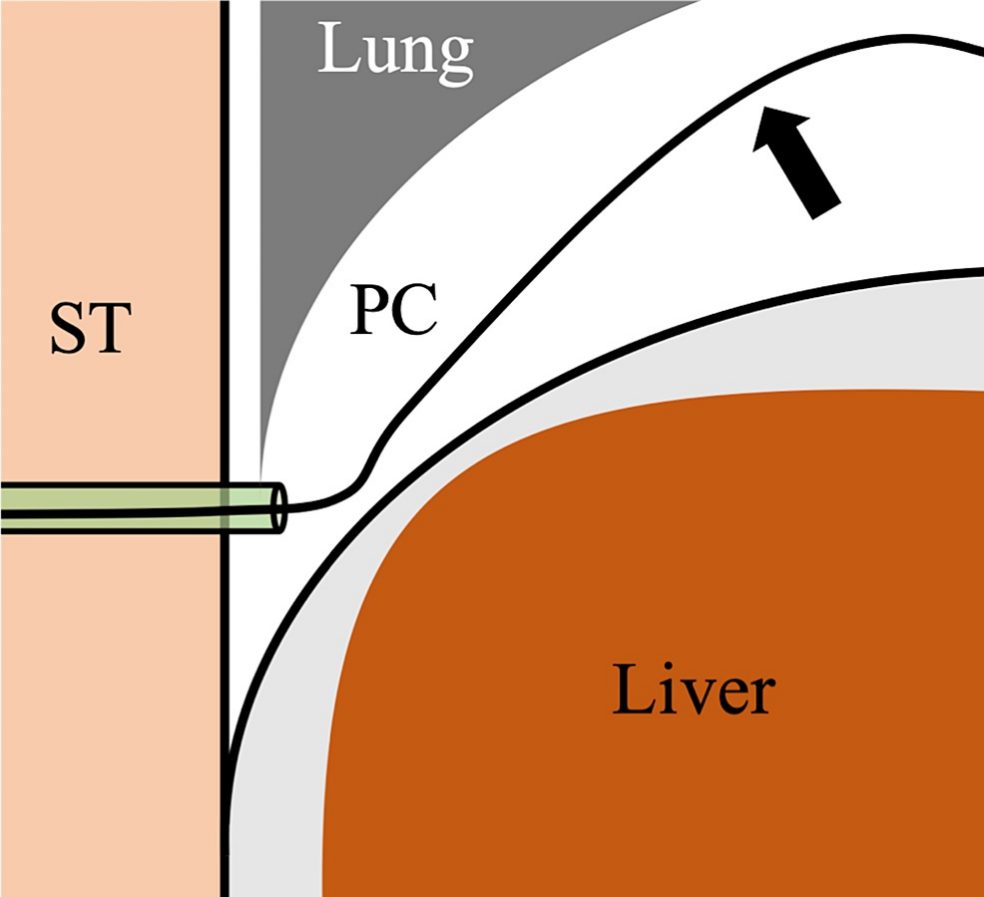
Schema of Seldinger’s technique to induce artificial pneumothorax (coronal image) The patient is instructed to inhale deeply to help advance the catheter tip further into the pleural cavity. Subsequently, a hydrophilic guidewire is inserted into the pleural cavity through the catheter (black arrow). ST: subcutaneous tissue, PC: pleural cavity.

**Figure 3:**
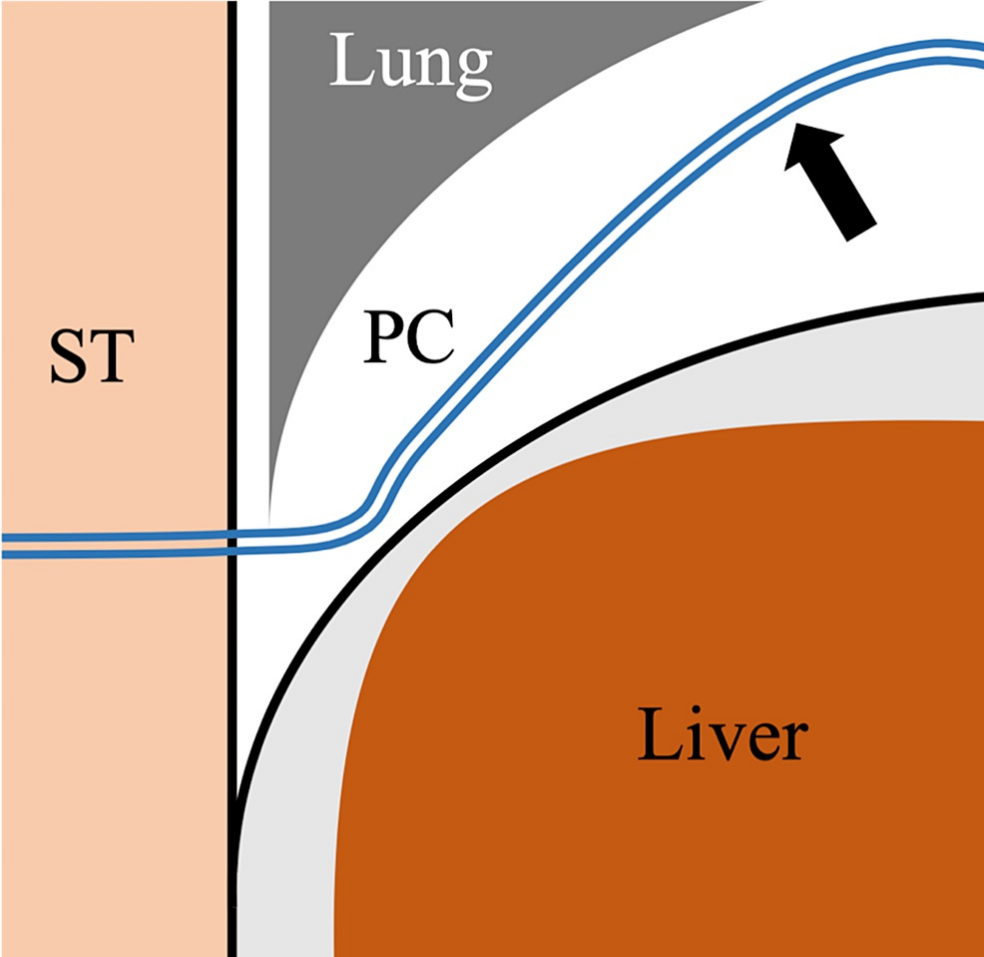
Schema of Seldinger’s technique to induce artificial pneumothorax (coronal image) Finally, small-diameter catheters are inserted into the pleural cavity over the guidewire to induce artificial pneumothorax (black arrow). ST: subcutaneous tissue, PC: pleural cavity.

**Figure 4:**
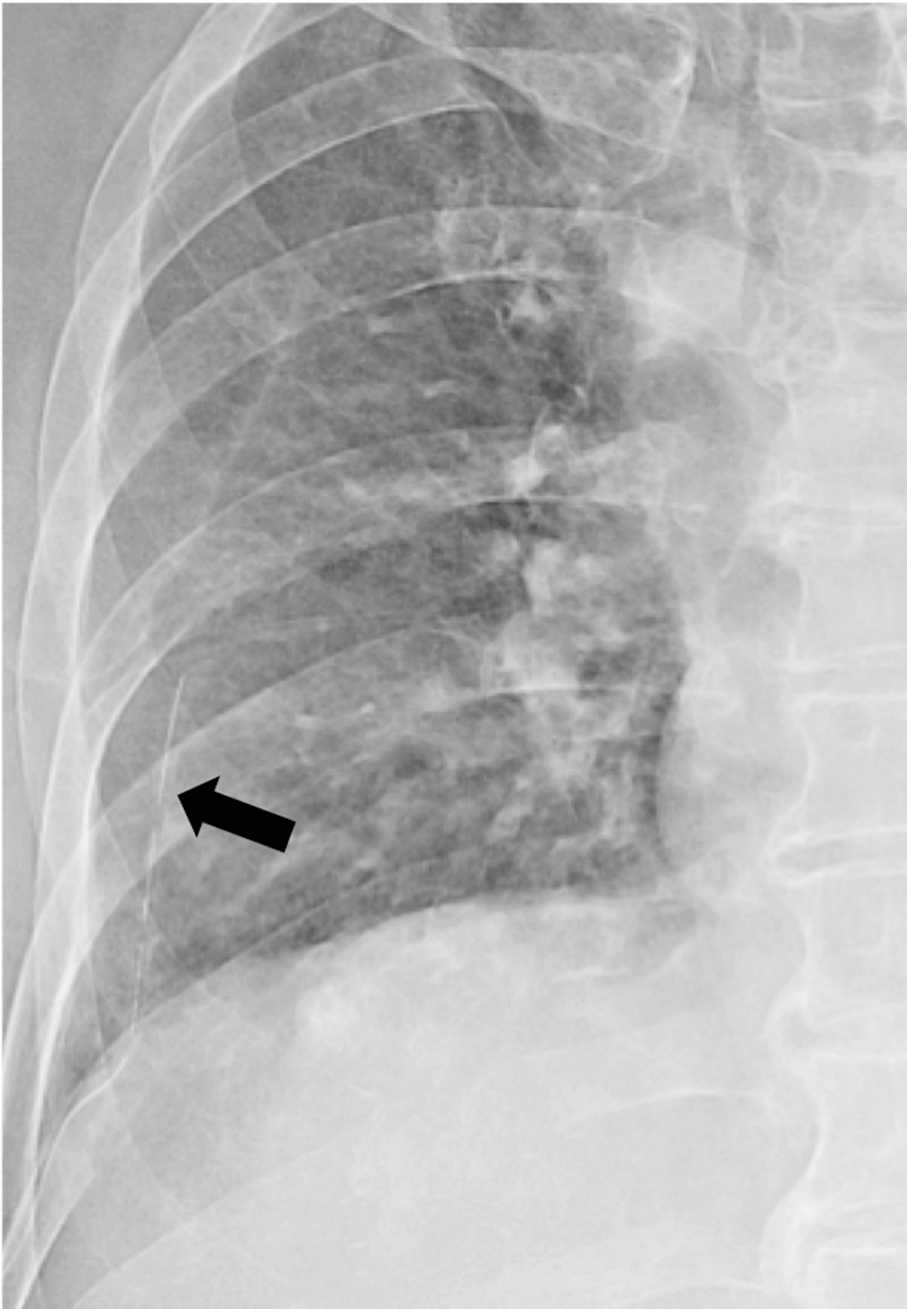
Image of a 78-year-old female patient who underwent radiofrequency ablation (RFA) with artificial pneumothorax for subdiaphragmatic hepatocellular carcinoma (HCC) Artificial pneumothorax was induced using Seldinger’s technique and a narrow catheter was inserted (black arrow).

**Figure 5:**
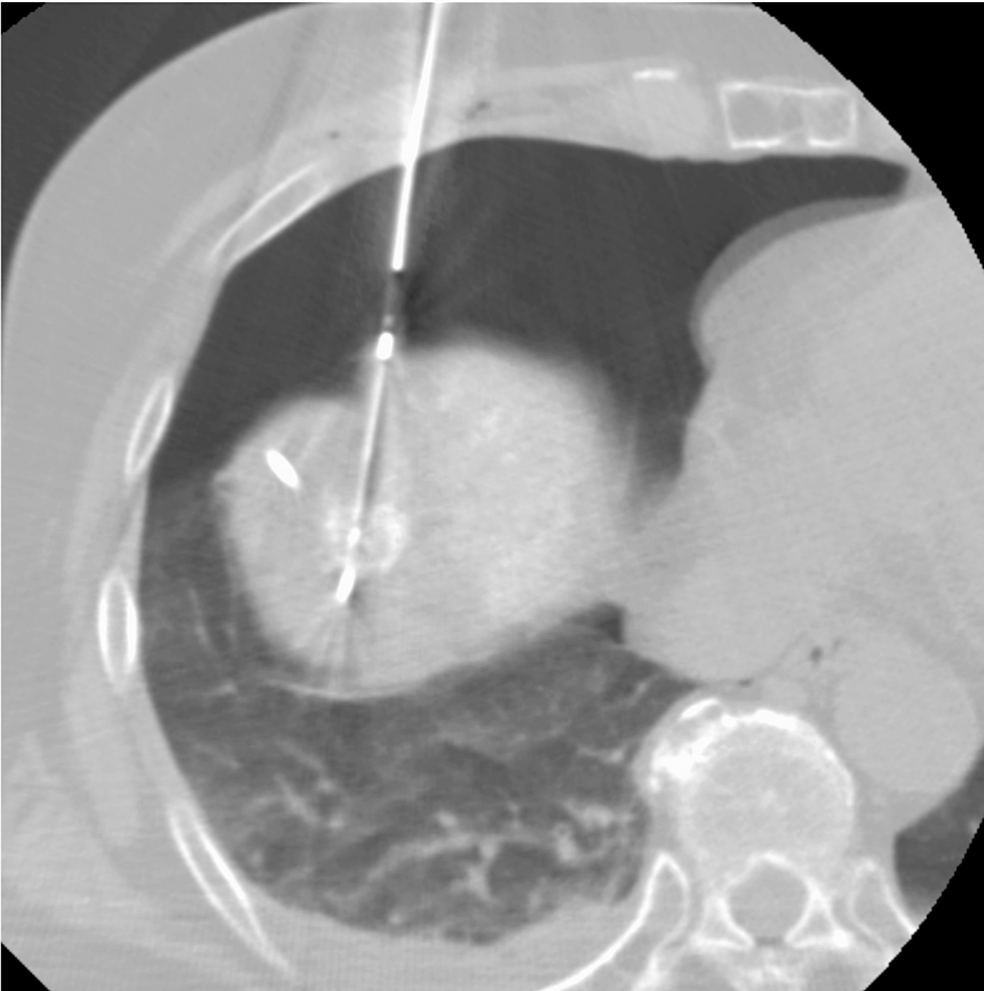
Images of a 78-year-old female patient who underwent radiofrequency ablation (RFA) with artificial pneumothorax for subdiaphragmatic hepatocellular carcinoma (HCC) With the artificial pneumothorax, the RFA for subdiaphragmatic HCC was successfully performed without lung injury.

All procedures were performed under local anesthesia with mild intravenous analgesics. The liver surface was punctured with an 18-gauge indwelling needle (Surflo, Terumo, Tokyo, Japan) under ultrasound guidance via the intercostal space in the inferior region of the thoracic cavity to avoid the lung parenchyma (Figure 1). The catheter was left in place and the patient was instructed to inhale deeply, thus moving the catheter into the pleural cavity. Subsequently, a hydrophilic guidewire (Radifocus Guidewire M; Terumo) was inserted into the pleural cavity through the catheter (Figure 2). Finally, small diameter (4- to 8-F catheter or soft tube) tubes were inserted into the pleural cavity over the guidewire, then artificial pneumothorax was induced and the percutaneous procedure was performed (Figures 3-5). After the percutaneous procedure, the gas was extracted as much as possible, and the catheter was removed.

Outcome measures

The following outcomes were evaluated in this study: procedure time (the time from local anesthesia to completion of the procedure), technical success (successful induction of artificial pneumothorax), clinical success (successful completion of the percutaneous procedure), and occurrence of complications, categorized according to the Clavien-Dindo classification [6]. The target lesions, catheter types, and types and amounts of gas were also evaluated. Additionally, the cases with indwelling catheters left in place after the procedure were evaluated.

Statistical analysis

Only descriptive statistics were performed (means and percentages).

## Results

Procedure details

The target lesions were 19 (76%) hepatocellular carcinomas, three (12%) mediastinal tumors, two (8%) renal cell carcinomas, and one (4%) adrenal tumor. The mean procedure time was 95.2 min (range: 25-160). The catheters used were 6-Fr percutaneous transhepatic cholangio-drainage (PTCD) tube in 15 (60%) cases, 8-Fr PTCD tube in five (20%) cases, 4-Fr seeking catheter in four (16%) cases, and 6.5-Fr seeking catheter in one (4%) case. The gases used were room air (18/25, 72%), CO2 (5/25, 20%), and mixed room air and CO2 (2/25, 8%). The amount of gas used was measured in 20 procedures, with a mean of 477.5 mL (range: 150-1000). The treatment details are summarized in Table 1.

**Table 1:** Details of percutaneous procedures with artificial pneumothorax of 25 patients HCC: hepatocellular carcinoma; RCC: renal cell carcinoma; PTCD: percutaneous transhepatic cholangio-drainage

Target Lesion	n (%)
HCC	19 (76%)
RCC	2 (8%)
Mediastinal Tumor	3 (12%)
Adrenal Tumor	1 (4%)
Procedure Time (minutes)	
Mean (range)	95.2 (25-160)
Type of Tube	
6F PTCD tube	15 (60%)
8F PTCD tube	5 (20%)
4F seeking catheter	4 (16%)
6.5F seeking catheter	1 (4%)
Type of Gas	
Room Air	18 (72%)
CO2	5 (20%)
Room Air + CO2	2 (8%)
Amount of Gas (ml, 20 cases are available)	
Mean (range)	477.5 (150-1000)
Technical Success	100%
Clinical Success	92%
Adverse events (Clavien–Dindo classification)	
Grade Ⅰ	3 (12%)
Grade Ⅱ	1 (4%)

Technical and clinical success

Artificial pneumothorax was successfully induced in all cases. Clinical success was achieved in 23 of 25 patients (92%). The remaining two patients underwent RFA and required the induction of a concomitant artificial pleural effusion to achieve clinical success. 

Complications

Three grade I and one grade II complication were observed, according to the Clavien-Dindo classification. All grade I complications were associated with biopsies and included two cases of pain and one of hemothorax that did not require treatment. However, in the patient with hemothorax, the catheter was left in place after the procedure for continuous drainage. The grade II complication, dyspnea, was associated with artificial pneumothorax and required oxygen therapy. 

## Discussion

Our results suggest that artificial pneumothorax induction using the liver-directed approach and Seldinger’s technique is highly feasible, with technical and clinical success rates of 100% and 92%, respectively. The maneuver is also safe, with no severe complications associated with the procedure.

Lin and Li reported that the technical and clninical success rates were 100% and 90.9%, respectively, in 11 cases of percutaneous mediastinal tumor biopsy with artificial pneumothorax. They used the two-step method to induce artificial pneumothorax [7]. Favelier et al. described a case of a percutaneous adrenal tumor with a biopsy performed by inducing artificial pneumothorax; they also used the two-step method to induce the pneumothorax [5]. No severe procedural complications were reported in either article.

Artificial pneumothorax can be applied to perform percutaneous ablations as well as biopsies [8-13]. A previous study on 26 RFA procedures for hepatocellular carcinoma reported that the technical success rate of artificial pneumothorax induction was 88.5%. Again, they used the two-step method to induce artificial pneumothorax, and no severe procedural complications occurred [4].

Artificial pneumothorax using the liver-directed approach and Seldinger’s technique has a low risk of lung injury since the pleural cavity is punctured where the lung parenchyma is absent. Additionally, inflation and deflation are straightforward phases compared to the two-step method due to the indwelling tube or catheter. Furthermore, the catheter can be used as a drainage device for pneumothorax and hemothorax if the latter occurs. Creating artificial ascites using Seldinger’s technique is feasible [14]. Our method is technically simple and similar to the creation of artificial ascites. 

A potential risk for hepatic hemorrhage exists because the puncture needle is advanced to the liver surface; however, such a risk is low because of the small diameter of the needle (18 G). Atwell et al. reported a low incidence of bleeding complications (0.5%) in their retrospective review of 3,636 percutaneous liver biopsies [15]. Since the puncture needle for the artificial pneumothorax is not inserted into the liver parenchyma, the rate of bleeding complication is expected to be lower than that of percutaneous liver biopsies.

This study has some limitations. First, it was a retrospective study conducted at a single institution with a small sample size. Second, the indwelling catheter and gas for the procedures were not standardized.

## Conclusions

Artificial pneumothorax induction for percutaneous procedures using the liver-directed approach and Seldinger’s technique may be a feasible and safe technique to avoid lung injury. However, further prospective studies with a larger sample size are warranted to determine the feasibility and safety of this maneuver.
